# Application of 3D printed titanium mesh and digital guide plate in the repair of mandibular defects using double-layer folded fibula combined with simultaneous implantation

**DOI:** 10.3389/fbioe.2024.1350227

**Published:** 2024-02-22

**Authors:** Shangbo Li, Lian Mi, Li Bai, Zijian Liu, Li Li, Yupeng Wu, Liqiang Chen, Na Bai, Jian Sun, Yanshan Liu

**Affiliations:** ^1^ The Affiliated Hospital of Qingdao University, Qingdao, China; ^2^ School of Stomatology, Qingdao University, Qingdao, China; ^3^ Zibo Infectious Disease Hospital, Qingdao, China; ^4^ Dental Digital Medicine and 3D Printing Engineering Laboratory of Qingdao, Qingdao, China

**Keywords:** digital design, 3D printed titanium mesh, 3D printed surgical guide, mandibular reconstruction, free fibular flap, immediate dental implant, occlusal reconstruction

## Abstract

Fibula transplantation plays an irreplaceable role in restoring the function and morphology of the defected mandible. However, the complex load-bearing environment of the mandible makes it urgent to accurately reconstruct the mandible, ensure the position of the condyle after surgery, and restore the patient’s occlusal function and contour. The intervention of digital design and three-dimensional (3D) printed titanium mesh provides a more efficient method and idea to solve this problem. Digital design guides the accurate positioning, osteotomy, and simultaneous implant placement during surgery, and 3D printed titanium mesh ensures stable condyle position after surgery, restoring good mandibular function. The double-layer folded fibula maintains the vertical height of the mandible and a good facial contour, and simultaneous implant placement can establish a good occlusal relationship. This study conducted a retrospective analysis of five patients with jaw defects who underwent digital fibula reconstruction over the past 3 years. It was found that the surgical protocol combining digital design, 3D printed intraoperative guides, 3D printed titanium mesh, free fibula flap, immediate implant, and occlusal reconstruction to repair jaw defects had more ideal facial appearance and biological function. It will provide a more reliable surgical protocol for clinical management of large mandibular defects.

## 1 Introduction

The mandible is a bone tissue with a “U-shaped” shape that plays an important role in chewing, swallowing, articulation, and other functions, and constructs the basic shape of the face. Trauma, tumors, infections, and other diseases often lead to mandibular defects, which have a serious impact on the patient’s life and reduce their quality of life (Chim et al., 2010). For patients with large mandibular bone defects, the common clinical treatment is segmental mandibular resection and vascularized free flap reconstruction. However, in the actual operation process, the repositioning of the mandibular stump, the restoration of the condyle position, the height and shape of the transplanted bone segment, and the restoration of the occlusal relationship are the key points and difficulties in mandibular reconstruction (Pamias-Romero et al., 2023). Therefore, it is urgent to develop new treatment methods to repair the mandibular bone defects, reconstruct a good facial contour, and restore the occlusal relationship for patients.

Since Hidalgo introduced free tissue transplantation to restore jaw defects (Hakim et al., 2015), vascularized fibula flaps have been widely considered the gold standard for mandibular reconstruction (Srivastava et al., 2022) due to their multiple advantages. When the vascularized upper layer of the double-layer fibula is suitable for implantation, the lower layer can effectively restore facial contour and provide better support for soft tissues (Li et al., 2023a). However, mandibular reconstruction is a complex task. Because the mandible consists of bilateral ramus and body, after the mandibular segmental resection, the mandibular osteotomy stump is unstable and loses the reference point for accurately reconstructing the bone defect. In addition, the condyle does not have an anatomical shape that can be used as a stable landmark, making it difficult to track its position and movement. Changes in the position of the condyle relative to the joint socket can lead to changes in the occlusion relationship (Tang et al., 2021). Surgical osteotomy destroys the three-axis relationship between the temporomandibular joint, occlusion, and muscles, making it necessary to spend a long time checking the position of the bone segments during mandibular reconstruction surgery. More importantly, it is difficult to determine the position of the condyle after osteotomy. Using preoperative Oral cone beam CT (CBCT) data, a titanium mesh consistent with the shape of the defect area is prepared through 3D printing. 3D printing titanium mesh is considered to be an effective way (Gao et al., 2019) to locate the position of the postoperative condyle because of its matching with the preoperative mandibular shape and low stress response. Relevant research has confirmed that 3D printed titanium mesh can ensure a more stable condyle position compared to titanium plates and reconstruction plates, and can also facilitate bone healing by inserting crushed bone fragments into the titanium mesh during surgery, which promotes early mandibular function (Li et al., 2023b).

Simultaneous implant surgery during the operation can restore the patient’s bite and chewing function early, reduce surgical trauma, and shorten the repair time (Srivastava et al., 2022). Simultaneous implant surgery can not only restore the patient’s chewing, articulation, and other functions early, but also play a positive role in slowing down the absorption of transplanted fibula, promoting bone tissue regeneration and reconstruction. Digital design is applied before surgery to print digital surgical guides, thus determining the range of osteotomy, surgical boundaries, and developing mandibular reconstruction schemes and methods. The surgical osteotomy guides are printed, which can significantly shorten the operation time, reduce surgical errors (Al-Sabahi et al., 2022), and accurately determine the range of osteotomy and implant implantation.

In recent related studies, the above-mentioned treatment methods have been introduced, but few cases include a complete treatment plan. A systematic approach to restoring the appearance and function of the mandible and facial appearance is currently lacking. In this situation, we provide a feasible solution for mandibular reconstruction, including digital design, 3D printing surgical guides, 3D printing titanium mesh, free vascularized fibula flaps, immediate dental implantation, and occlusal reconstruction.

## 2 Patients and methods

This study received approval from the Ethics Committee at the Affiliated Hospital of Qingdao University, Qingdao (ethics number, QYFY WZLL 28268). This study was conducted at the Affiliated Hospital of Qingdao University from January 2020 to October 2023. Patients undergoing digital design-guided double-layer folded fibula transplantation and concurrent implant surgery in the department of oral and maxillofacial surgery were included in the cohort. The cohort included three females and two males. All patients suffered from extensive mandibular defects due to tumors, two of whom had mandibular defects after resection of malignant tumors and required restoration of facial appearance. All surgeries had completed preoperative digital surgical design; 3D printed titanium mesh was used to guide double-layer folded fibula transplantation and concurrent implant surgery to complete mandibular reconstruction. The dual-team approach was maintained throughout the study.

### 2.1 Preoperative digital design, 3D printing osteotomy guide plate, and 3D printing titanium mesh preparation

Before surgery, all patients underwent lower limb arteriovenous color ultrasound evaluation of lower limb blood flow, routine pre-operative 3D CT scanning of the fibula (SOMATOM Force CT, slice thickness 0.625 mm) and CBCT (KaVo i-CAT 17-19) examination of the jaw. 3dMD (3dmdface.t system, England) was used to complete pre-operative 3D facial scanning. The obtained jaw, fibula, and facial data were analyzed using Mimics 17.0 software (Materialise, Leuven, Belgium). The software is used to simulate the resection of the primary tumor, fibula osteotomy, mandibular reconstruction scheme design, osteotomy guide plate and implant placement position design. In the design, the osteotomy guide plate and reconstruction guide plate always use the same set of screw trajectories to minimize errors and bone block displacement. After mandibular reconstruction, the 3D facial data were fitted to evaluate the degree of facial contour change. After the surgical design is completed, the designed guide plate is printed using a 3D printer (LITE-450HD), at the same time, the 3D titanium mesh (stema Germany) is printed using the above data.

### 2.2 Tumor resection and mandibular reconstruction

After the surgical team completes the preoperative digital design, a team of doctors cuts the skin and muscle layer, and places the osteotomy guide plate according to the preoperative design. According to the preoperative virtual surgical design, partial mandibulectomy and tumor resection are performed. Another team of doctors extracts the fibula with a vascular pedicle. After complete tumor resection, two sets of recipient vessels are prepared to complete fibula transplantation. At this time, the repair team enters the operation room and determines the implant placement position and depth under the guidance of the preoperative design implant guide plate. The simultaneous implant surgery is completed on the transplanted fibula, and the incision is closed. After the surgery, the patient will be sent to the intensive care unit (ICU) for close observation of the general condition and respiratory condition. The patient is transferred back to the general ward on average within 2–3 days after the surgery. All patients need to receive an enteral fluid diet combined with intravenous nutritional support treatment within 1 week after the surgery. Subsequently, the transition to an oral fluid diet, semi-liquid diet, and normal diet is gradually carried out. The patient is expected to return for follow-up surgery for the second stage of implant treatment 6 months after the surgery. Each patient was followed up for at least 1 year, during which surgical and medical complications were recorded. A patient satisfaction questionnaire was designed to provide subjective evaluations of chewing, articulation, and facial appearance, which were divided into three levels: very satisfied, satisfied, and dissatisfied. To determine objective evaluations, facial symmetry, facial height, articulation, chewing, swallowing, mandibular movement, and other factors were evaluated. A Likert scale was used for scoring (where 1 = very abnormal and 5 = completely normal).

## 3 Results

The five patients in our study ranged in age from 18 to 47 years, with an average age of 29.4 years. Two of the patients had previously suffered from oral squamous cell carcinoma, and are now 7 and 12 years after tumor resection, respectively. Three patients had mandibular ameloblastoma. All five patients underwent bilateral fibular folding for repair, with fibular lengths ranging from 109.15mm to 191.42 mm (average value of 138.25 mm). The average number of implants placed during the same period was 3.6. The anastomosis involved recipient arteries, including the facial artery, the superior thyroid artery, and the lingual artery; recipient veins included the facial vein, the superior thyroid vein, the internal jugular vein, and the external jugular vein, and donor arteries and veins included the fibular artery and the fibular vein. The duration of these surgeries ranged from 500 to 830 min, with an average of about 640 min, and no vascular crisis or pedicle fibula flap necrosis occurred after surgery. The average follow-up time for the 5 patients was 15.8 months (range: 12 months–19 months). Patient satisfaction assessment showed that 3 patients were very satisfied with the appearance after surgery, 2 patients were satisfied with the appearance after surgery, and the completion time of the repair for the 5 patients was 11.2 months (range: 9 months–15 months). After the operation, the facial CBCT soft tissue data was mirror-fitted, and the maximum difference value of bilateral soft tissue after fitting was 4.45 mm on average. What is impressive is that all 5 patients recovered their chewing function within 1 month after surgery, and had a good facial appearance (Table 1).

**TABLE 1:** Patient data.

Case no.	Age (Y)	Sex	Pathogenesis	Time of operation (min)	Length of the fibula (mm)	Repair completion time (month)	Follow-up period (month)	Likert scale scores	Length of fit (mm)
								(10-50)	
1	18	F	AME	640	134.76	11	18	43	3.46
2	23	M	SCC	500	117.38	9	14	47	1.93
3	32	F	AME	830	191.42	15	16	46	7.11
4	47	M	SCC	710	138.54	10	19	43	6.89
5	27	M	AME	520	109.15	11	12	44	2.88
Average	29.4	—	—	640	138.25	11.2	15.8	44.6	4.45

AME:ameloblastoma SCC:squamous cell carcinoma.

Length of fit: the soft tissue mirror was fitted to the maximum gap length.

### 3.1 Case presentation

**FIGURE 1:**
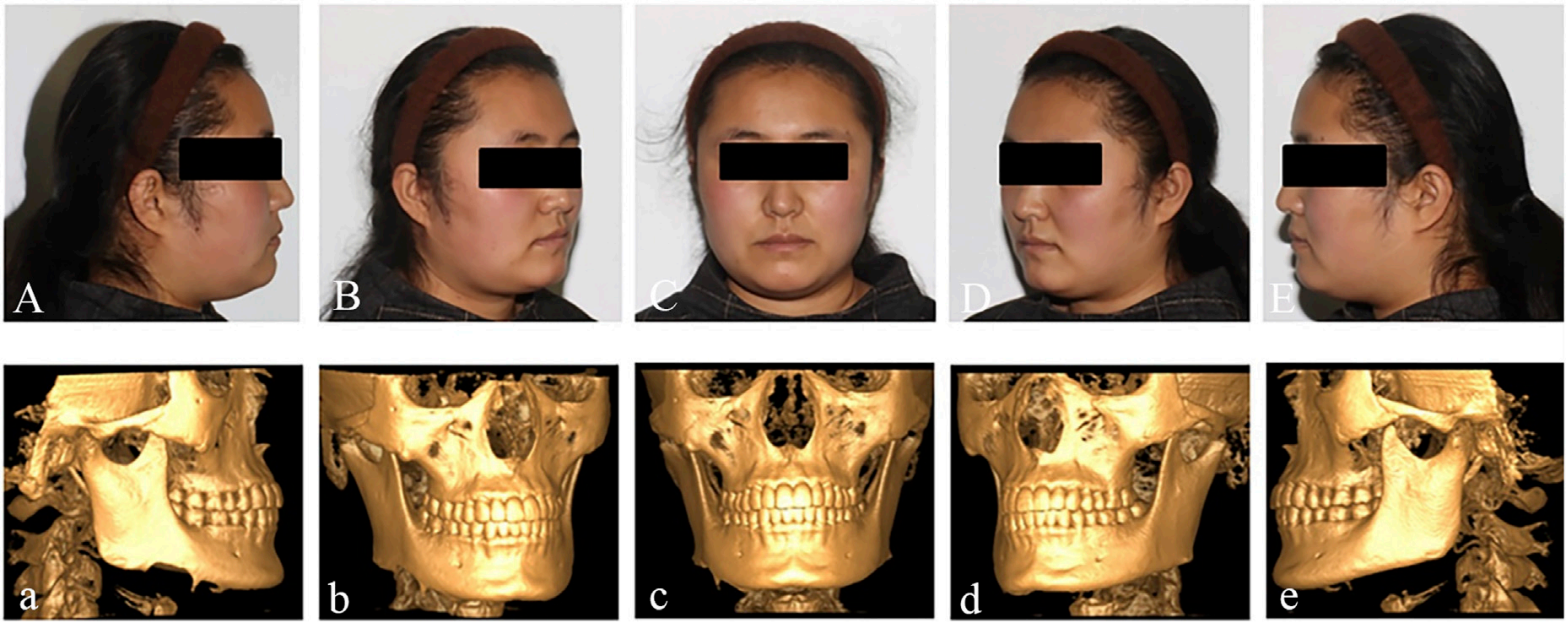
Preoperative facial photos and CBCT.

A 27-year-old female patient presented to the Affiliated Hospital of Qingdao University with a 4-month history of swelling in the right lower posterior region. The outpatient doctor removed the 47 and performed a pathological examination, which revealed that the patient had an ameloblastoma in the right mandible (Figure 1). In addition to the mandibular tumor, the patient also suffered from schizophrenia for 8 years and was taking oral medication. The patient’s condition is now under good control.

**FIGURE 2:**
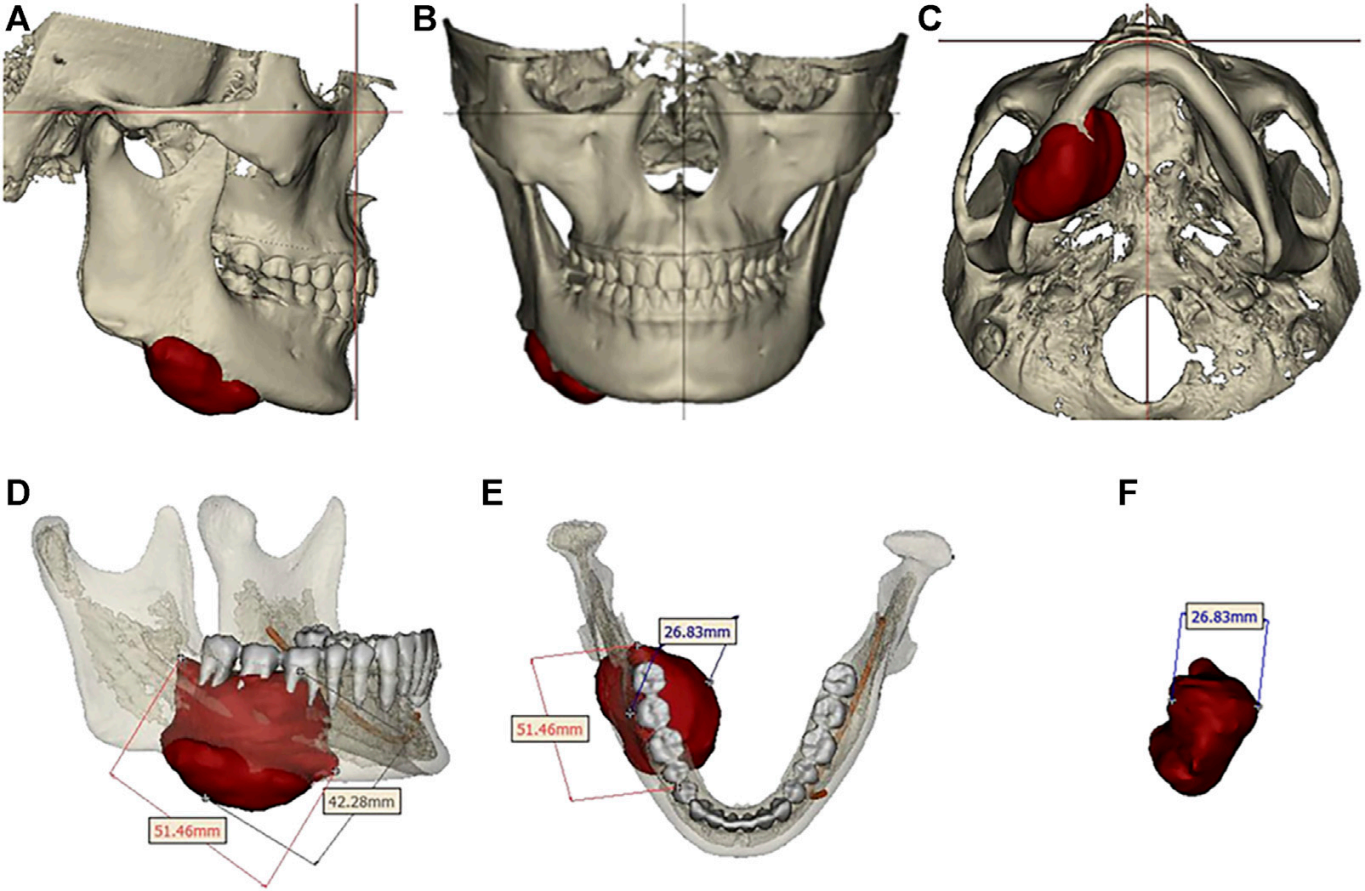
Preoperative tumor size and extent of involvement.

Preoperative examination revealed asymmetry in bilateral oral and maxillofacial regions, with a slightly longer height of the right mandibular ramus than the left, and a 47 missing. A bone swelling could be felt on the lingual side of the right mandible, with a dark red fistula on the surface. A slight squeeze showed a small amount of yellow liquid exudation. The opening degree was 4.5cm, and the opening type was normal. The occlusion relationship was acceptable. No obvious enlarged lymph nodes were felt in the bilateral submandibular region and neck. Complete CBCT showed that the tumor size was 51.46 mm × 42.28 mm × 26.83mm, involving 44-47 teeth, and the right cortical bone of the mandibular body was damaged. The right mandibular nerve tube was damaged (Figure 2). Complete 3DMD was performed before surgery, and CT data was fitted with facial data.

According to the patient’s condition and needs, the surgical plan was formulated as a comprehensive treatment strategy encompassing mandibular tumor excision, mandibular reconstruction, and dental rehabilitation. This encompassed the utilization of free vascularized bilateral fibula grafting for the reconstruction of mandibular defects, 3D-printed titanium mesh reconstruction of the mandible, prompt implant placement, and the utilization of implant-supported fixed dentures for restoration.

**FIGURE 3:**
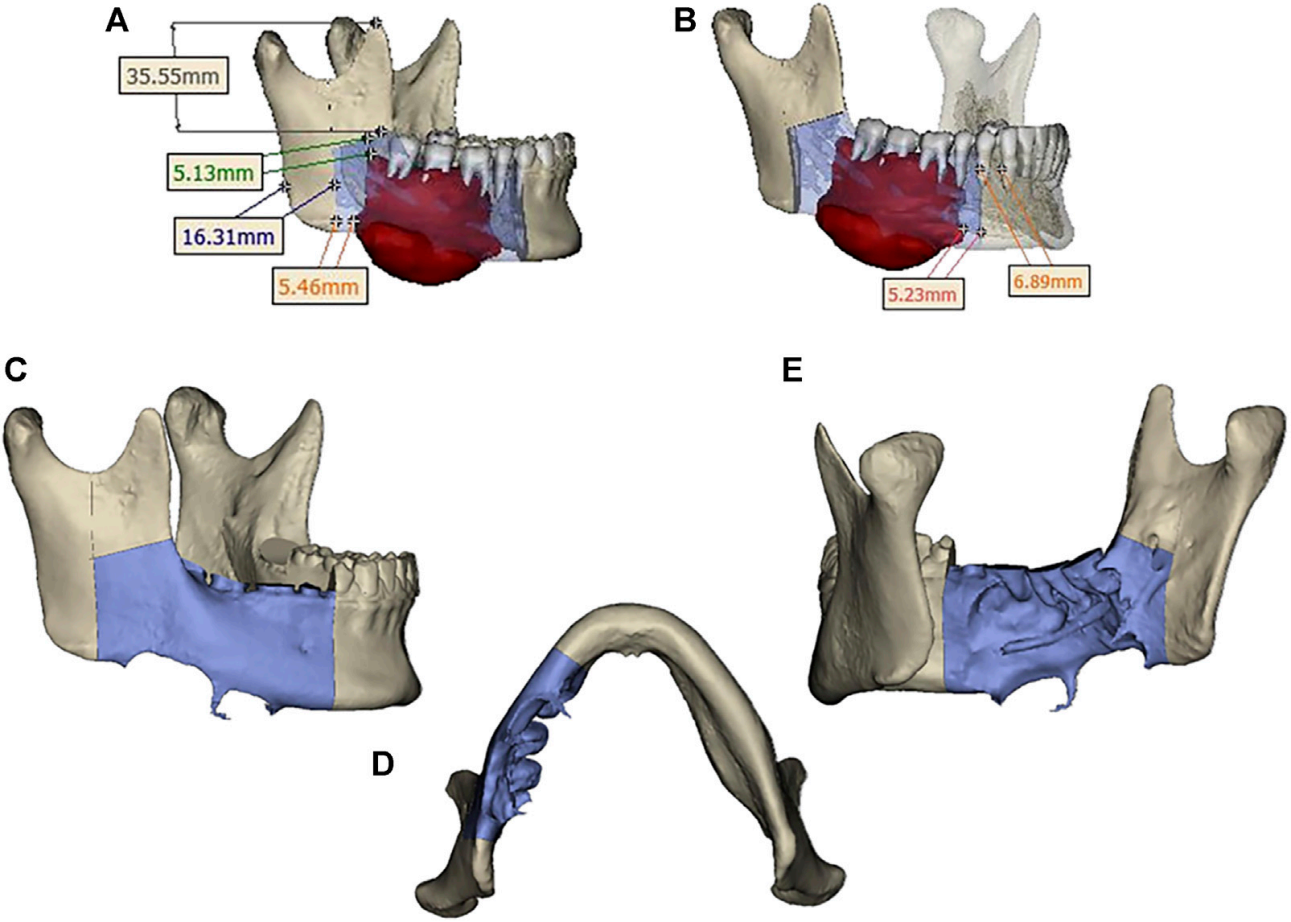
**(A)** Osteotomy range of the right mandibular ramus. **(B)** extent of mandibular body osteotomy. **(C, D, E)** Three-dimensional view of the extent of mandibular osteotomy.

**FIGURE 4:**
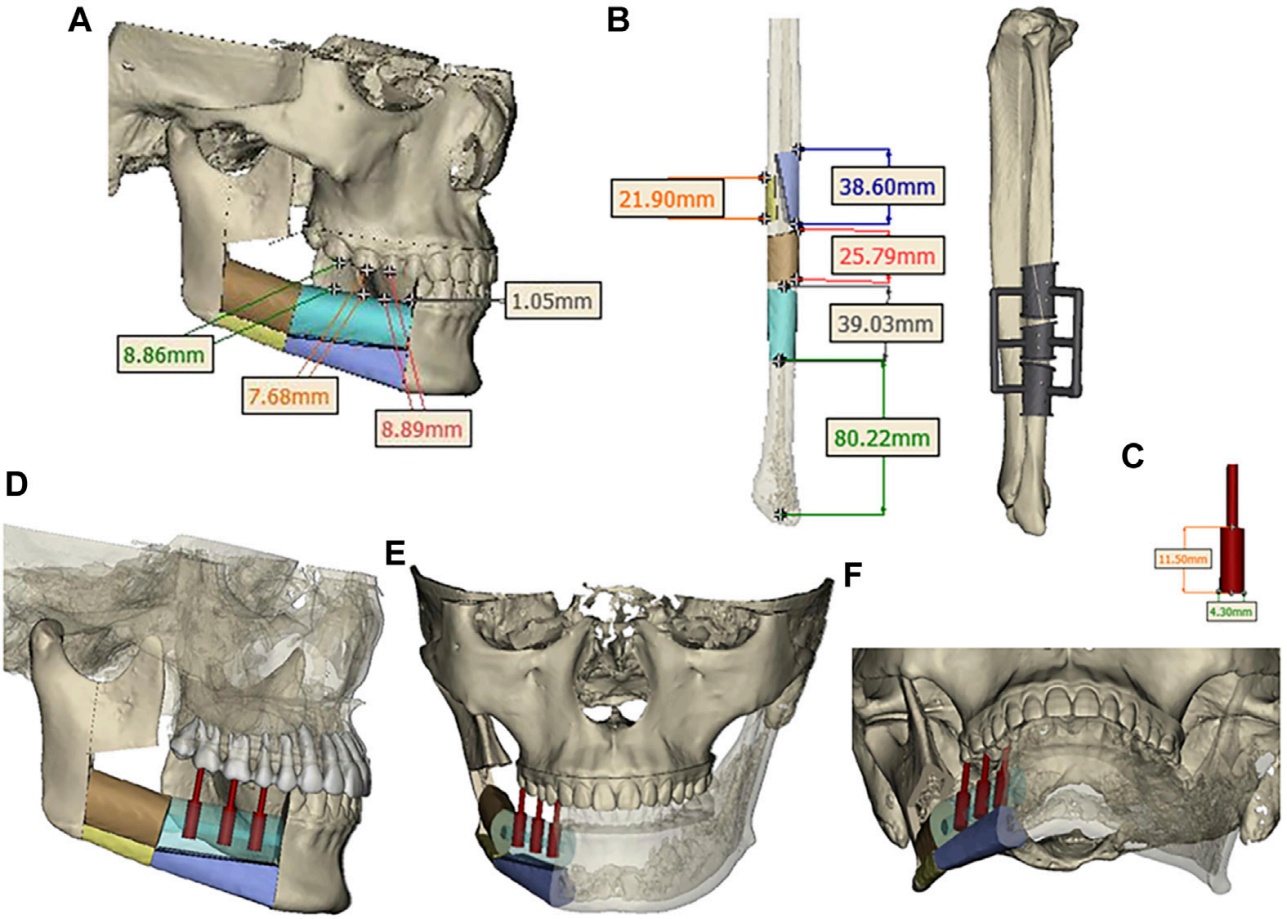
**(A)** Segmentation and arrangement of fibular segments. **(B)** fibular osteotomy and osteotomy guide plate. **(C)** Implant size. **(D, E, F)** Three-dimensional view of implant placement.

Design a digital surgical plan for the patient using Mimics software. The mandibular osteotomy range was: the distance from the right mandibular ascending branch osteotomy line to the tumor was approximately 5.46–5.13mm, approximately 16.31 mm from the posterior edge of the right ascending branch, and approximately 35.55 mm from the right coronoid process. The right body osteotomy line was located at 44 teeth, approximately 5.23 mm from the tumor, and approximately 6.89 mm from the root of 43 teeth. Preoperative preparation of 3D printed mandibular body osteotomy guides, chin-support osteotomy guides, and chin-tooth support osteotomy guides were used to accurately guide the osteotomy range. The fibula was taken from 80.22 mm above the left lateral malleolus, with a total length of approximately 109.15 mm. The fibula was divided into four segments, placed in two layers, with the upper layer pedicled and the lower layer free. The proximal end was placed from the right mandibular angle, and the distal end was close to the chin. The lateral side of the fibula corresponded to the lateral side of the jawbone. After fitting with the preoperative digital design, a 3D titanium mesh was printed to fix the fibula segment. The distance between the transplanted fibula and the maxillary cusp was approximately 7.68–8.89 mm (Figures 3, 4).

According to the preoperative design plan, the mandibular tumor was completely removed under the guidance of the osteotomy guide plate, the vascularized bilateral fibula was transplanted, and the mandible was reconstructed with 3D-printed titanium mesh. During the operation, three implants were implanted in the transplanted fibula. First, the surgeons were divided into two groups and performed the surgery simultaneously. One group cut the left vascularized fibula, while the other group cut part of the right mandible. When cutting the left vascularized fibula, firstly, the tourniquet was used to stop the bleeding, and the skin, subcutaneous tissue, deep fascia, and muscles were separated along the preoperative design to find the fibula. Avoid injury to the superficial peroneal nerve and anterior tibial blood vessels during the operation and the long and short muscles on the fibula were cut. After accurately placing the fibula osteotomy guide plate in the designed position, a wire saw was used to cut the mandible at a distance of 8 cm above the lateral malleolus, and then the required fibula segment was cut. The fibular vascular pedicle and posterior tibial artery branch of about 3 cm proximal to the fibula were preserved and cut, and the vascular anastomosis was prepared. The tourniquet was loosened and the surgical wound was closed. Wash the proximal artery and two veins with heparin saline to separate them. The repaired fibula segment is wrapped in saline gauze for backup.

**FIGURE 5:**
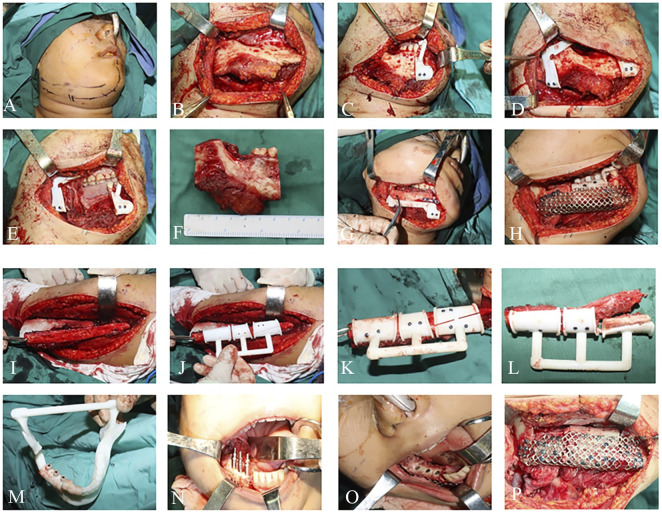
Intraoperative photographs: **(A–F)** Mandibular tumor resection. **(G–L)** Mandibular reconstruction. **(M–P)** Implant placement.

Mandibular partial resection and reconstruction. The intraoral incision is designed from the center of the lower lip to the 33 gingiva along the gingival margin. The extraoral incision is designed from the center of the lower lip outward to the submental region and continues along the right mandibular inferior margin for 2 cm to the right mandibular angle. After accurately placing the fibula osteotomy guide plate in the predetermined position, the skin and superficial fascia are incised, the muscles are separated, and the mandible is exposed. The 44, 45, 46, and 48 teeth are extracted, and the osteotomy guide plate is placed on the mandibular body and chin, respectively, for precise osteotomy. The right mandible and tumor are completely removed, and the specimen is sent for pathological examination. Under the guidance of the guide plate, the free fibula is divided into four segments. Under the guidance of the titanium mesh and positioning guide plate, the four segments of fibula are placed at the reconstruction site of the mandible and then fixed with titanium mesh and titanium screws. Under the operating microscope, the superior thyroid artery is anastomosed with the fibular artery, and the internal jugular vein is anastomosed with two fibular veins. The implant guide plate is placed on the fibula, and the positioning is performed using a spherical drill under the guidance of the guide plate. The pioneer drill is used to prepare the implant site, and determine the direction and depth. After detecting the planting site with a pointer, the planting site was enlarged with a pioneer drill. Finally, implants 45(Nobel Active 4.3 × 11.5 mm), 46(Nobel Active 4.3 × 11.5 mm), and 47(Nobel Active 4.3 × 11.5 mm) were implanted into the fibula and covered with screws. The muscles were anastomosed to the soft tissue of the floor of the mouth, and the incision was closed layer by layer. After surgery, the patient’s facial appearance recovered well (Figure 5).

**FIGURE 6:**
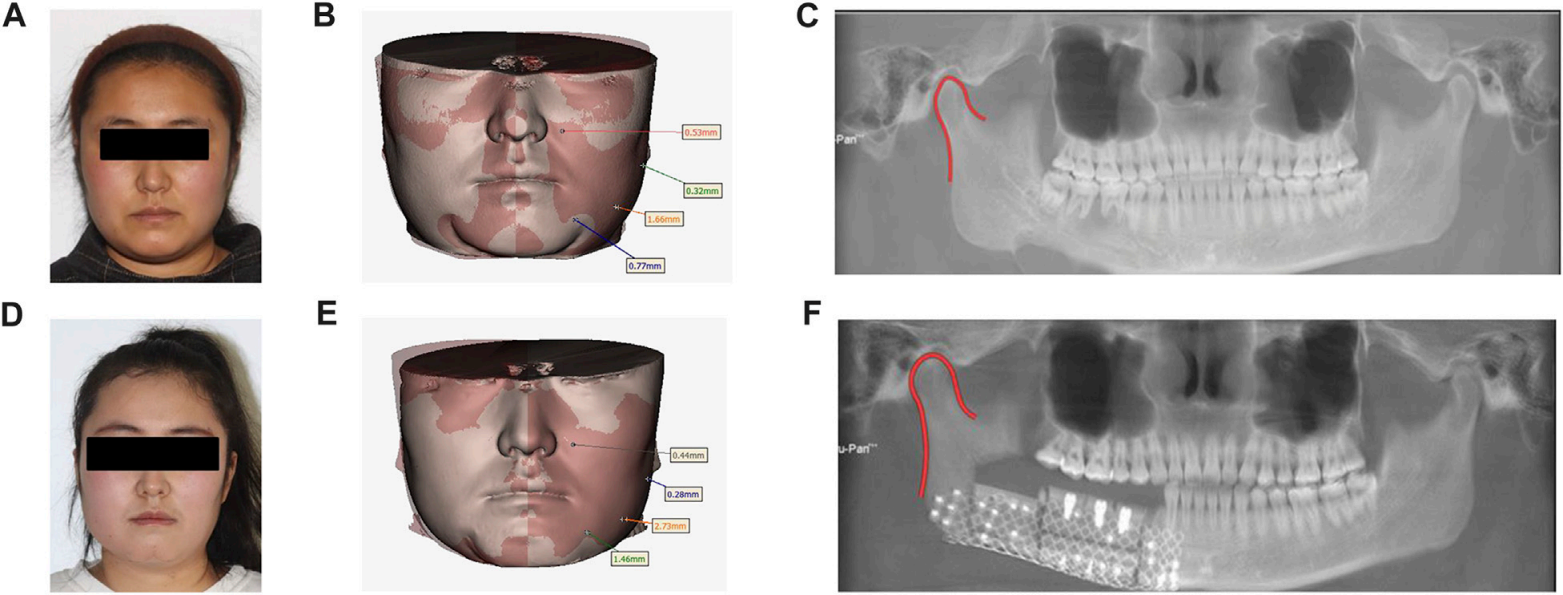
Preoperative and postoperative contrast: **(A)** Preoperative facial features. **(B)**. Preoperative facial soft tissue mirror image fitting was performed. **(C)**. Preoperative panoramic radiographs. **(D)**. Postoperative facial features. **(E)**. Postoperative facial soft tissue mirror fitting. **(F)**. Postoperative panoramic radiographs.

The patient returned for a 1-month follow-up after surgery and was seen to have stable occlusion, good recovery of the surgical area, and a generally symmetrical facial appearance. The patient returned for a 3-month follow-up after surgery, and the appearance of the maxillofacial region was well-restored, with basic symmetry on both sides; the intraoral occlusion was stable. CBCT showed a first-stage healing of the implant, the condyle was accurately positioned, ang the bone block was well-aligned, with no absorption or displacement, and the patient could open their mouth early to eat and restore the occlusal function (Figure 6).

After a 6-month follow-up following surgery, the implants have healed well. However, the mucosa after fibula grafting is non-keratinized, which may increase the risk of peri-implantitis. When the second stage of implant surgery is performed, we plan to increase the keratinized mucosa using acellular allogeneic dermis (ADM). During the surgery, the mucosa was incised, the medium-thick skin flap was sharply separated, and some connective tissue and periosteum were retained to expose the screw cover. The screw cover was replaced with a healing abutment (45, 46 were 5.0 × 5.5mm, 47 was 5.0 × 3 mm), and ADM was fixed to the sides of the abutment, adhering to the periosteum and sutured to the medium-thick gingival flap and periosteum. The ADM was stabilized using iodoform gauze stuffing. A follow-up after 1 month revealed that the ADM did not properly bond with the non-keratinized mucosa on the buccolingual side, so we decided to perform a free gingival graft (FGG) to widen the keratinized gingiva. The free gingival on the 23–26 palatal side was removed and an absorbed gelatin sponge was used to fill the donor area of the keratinized gingiva, with sutures fixed to the sponge. The corresponding position of the free gingiva was drilled and inserted into both sides of the healing abutments and sutured. Finally, a hemostatic plate was made to compress and protect the keratinized gingiva donor area.

Two months after the FGG surgery, the gum healed well and entered the final stage of implant restoration. 44, 45, and 46 were selected for fixed bridge crown restoration, and 47 was selected for single crown restoration. We selected a window-type personalized tray and corresponding implant transfer rod made of silicone rubber to take the final impression. Due to the stable occlusion relationship of the patient’s left teeth, we sent the model and recorded occlusion relationship to the processing factory to produce a resin temporary tooth. To ensure that the resin temporary tooth has stable occlusion during centric occlusal movement and lateral jaw movement, and improve chewing efficiency. After wearing the temporary tooth for 2 months, the patient’s temporomandibular relationship was comfortable and the chewing function was good.

**FIGURE 7:**
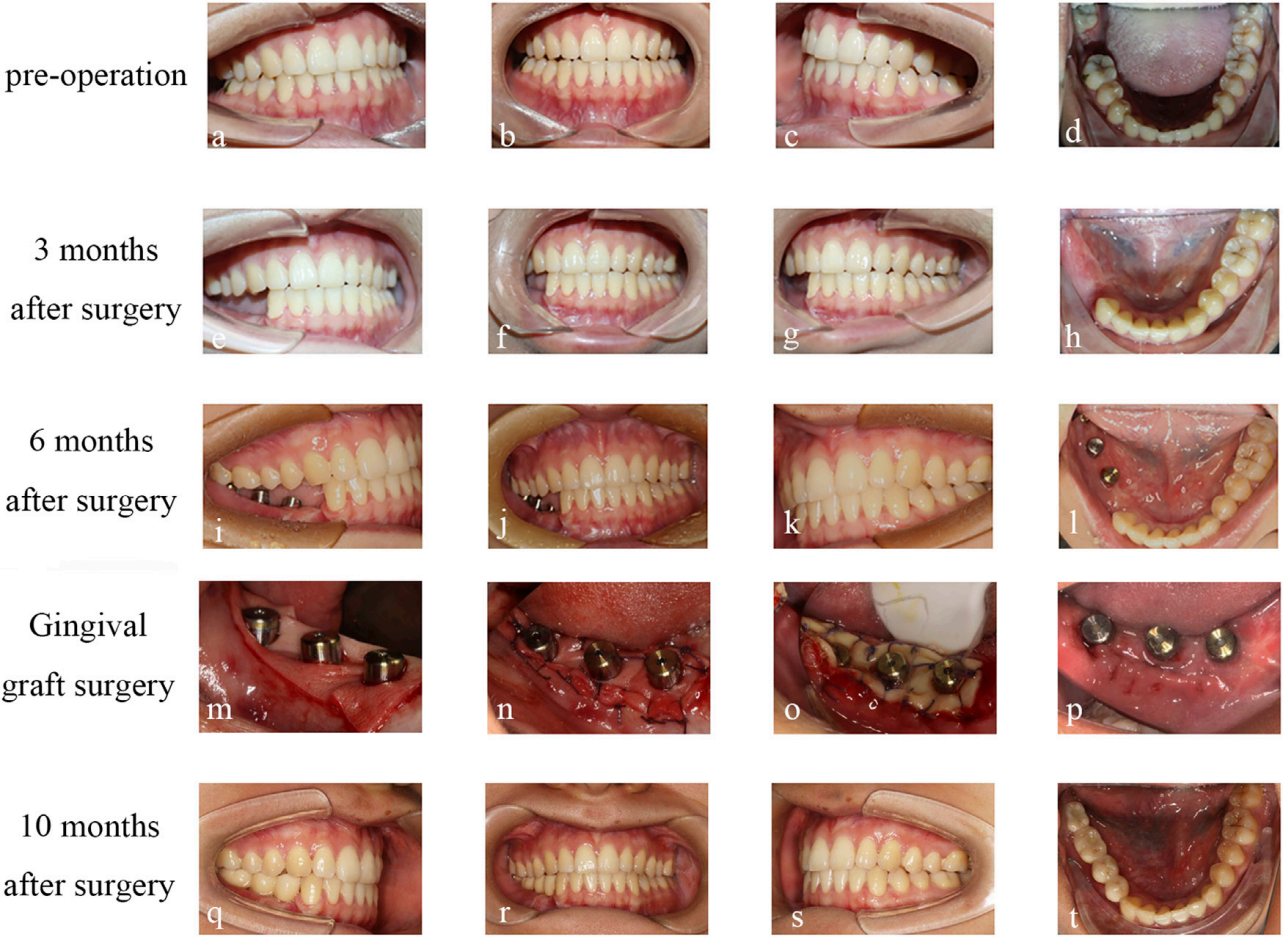
Condition of occlusion: **(A–D)** Preoperative occlusion. **(E–H)** occlusion at 3 months after operation. **(I–L)** occlusion at 6 months after operation. **(M, N, O)** ADM transplantation. **(P)** FGG transplant follow-up 2 weeks after surgery. **(Q–T)** occlusion was performed at 10 months after operation.

Finally, the final restoration stage was carried out, with the installation of personalized abutments in 45 and 46, and the installation of the original abutment-integrated crown in 47. The final restoration in 44, 45, 46, and 47 selected zirconia all-ceramic crowns. Through facial comparison, the patient’s appearance was effectively rehabilitated. CBCT soft tissue mirror fitting showed no significant changes in the facial soft tissue before and after surgery, and the maximum difference in soft tissue between the affected and healthy sides after surgery was only 2.88 mm. Imaging comparison of condyle position showed no significant displacement of the condyle. The facial scan results indicated that the patient’s appearance was effectively rehabilitated through mandibular reconstruction and dental implant placement. The patient expressed profound satisfaction with the final outcome (Figure 7).

## 4 Discussion

Mandibular reconstruction and dental restoration are complex tasks that are influenced by multiple factors and involve technologies from different fields. Currently, there are still some inevitable limitations in digital design and 3D printing technology (Al-Sabahi et al., 2022). The design and 3D printing of preoperative guides consume a lot of time, which may delay the treatment opportunity. The accuracy of CT scanning may be affected, as CT scanning cannot accurately capture the occlusal anatomical structure, which may also affect the final surgical design effect. Design errors of surgeons and engineers are still inevitable (Anuja et al., 2011). The success of treatment depends on the close cooperation of multidisciplinary teams including oral and maxillofacial surgery, oral implantology, prosthetics, and computer engineers.

After tumor resection, segmental mandibular defects are accompanied by many physiological problems, including poor occlusal function, changes in mandibular morphology, changes in mandibular motion trajectory, language dysfunction, chewing and swallowing difficulties, and facial deformities. Mandibular reconstruction not only restores the continuity of bone tissue and facial appearance, but also restores normal chewing, swallowing, and vocalization functions to achieve coordination between oral function and facial appearance (Moiduddin et al., 2019). The repair of segmental mandibular defects is of great significance in improving the quality of life of patients.

The transfer of a double-layer folded vascularized fibula flap can construct a good vertical height of the face, slow down the absorption of the fibula, and provide sufficient and safe bone tissue for simultaneous implantation. Simultaneous implantation can provide patients with good occlusal relationships and mandibular functions (Tumuluri et al., 2023), and restore the patient’s chewing function as early as possible.

The goal of mandibular reconstruction is to restore chewing, speech function, and acceptable appearance. Using digital immediate implant restoration, dental implants are implanted into the transplanted fibula and can be restored using removable dentures or fixed dentures. A large number of studies have shown that 3D printing digital implant guides combined with preoperative rational planning of implant position can achieve precise and minimally invasive surgery (Londono et al., 2018). In previous clinical studies, most mandibular reconstructions required an interval of about 1 year before undergoing implant surgery, delaying the repair treatment cycle and seriously affecting the patient’s quality of life. Immediate implant effectively solves the above problems (Miljanovic et al., 2022). Due to the precise preoperative planning of the implant position, the implant is embedded into the fibula after immediate implantation, which can avoid infection from saliva and microorganisms in the oral cavity and form good osseointegration.

In addition, soft tissue reconstruction has a significant impact on the prognosis of implants with fibula grafts. In general, after fibula grafting, the peri-implant soft tissue does not adequately replicate keratinized mucosa, which increases peri-implantitis risk (Nakaue et al., 2020). For this reason, we may need to perform soft tissue reconstructions to improve the condition of the soft tissues around the implants. Compared with FGG, ADM has the advantages of a single surgical site, no need to create a second surgical area, less postoperative pain and discomfort, better aesthetics, and integration with surrounding tissues. Patients have less psychological burden and the surgical trauma is smaller. ADM does not have cellular and tissue-inducing abilities, and the repair process mainly relies on the migration of adjacent tissue cells to integrate with surrounding tissues (Shan et al., 2022). It is possible that the excessive pressure of the tamponade affected the blood supply, resulting in the inability of the ADM graft to completely fuse with the fibular mucosa. Therefore, in the subsequent treatment of FGG, we made improvements to this part. Besides, it may be related to the position of the implant, the size and thickness of the gingiva in the donor area, the adaptability of the graft, the preparation of the recipient area, and the oral hygiene maintenance around the implant (Shan et al., 2022). Clinicians should strengthen the guidance of patients’ oral hygiene maintenance and professional implant maintenance treatment to reduce the risk of peri-implantitis.

Despite the complex anatomical structure of the mandible, 3D virtual surgical planning provides a platform for surgeons and engineers to collaborate to develop surgical plans. During the operation, the precise intraoperative osteotomy position is determined to minimize the extent of bone removal and preserve important anatomical landmarks. Currently, digital design and 3D printing have been widely used in mandibular reconstruction, maxillofacial trauma, implantology, temporomandibular joint reconstruction, orthognathic surgery, and other fields, providing powerful technical support for preoperative diagnosis, surgical plan development, disease analysis, and surgical implementation (Moiduddin et al., 2019) in oral and maxillofacial surgery. 3D-printed titanium mesh is based on digital design and maximally adapts to the mandible contour. With the help of digital design and 3D-printed surgical guides, surgeons minimize the extent of osteotomy and reconstruct the mandible contour before the disease to the greatest extent possible.

It can be seen that the advantages of digital surgical design and 3D printing technology are as follows: 1) digital design transfers surgical design to the preoperative stage, reduces the cost of trial and error, simplifies surgical steps, and shortens surgery time (Succo et al., 2015), especially for the repair of large mandibular defects (Liu et al., 2014). 2) Full digital design has good shaping effects in repairing mandibular defects with double-layer folded fibula muscle flaps (Gao et al., 2021), establishing good occlusal relationships and mandibular functions. 3) In conventional surgery, repeated grinding of fibular fracture ends by doctors can increase the damage to the fibular blood supply and surrounding tissues. The use of 3D technology can avoid these problems, thereby reducing intraoperative bleeding (Anuja et al., 2011). 4) Digital design can more accurately guide implant placement sites and depths, providing a good foundation for early repair and reconstruction (Schepers et al., 2013). 5) Compared to titanium plates or reconstruction plates, 3D printed titanium mesh can implant crushed bone fragments into bone defect areas during surgery (Shan et al., 2015), promote early healing of the bone tissue, and have excellent mechanical properties (Li et al., 2023c), ensuring accurate positioning of the condyle, and the titanium mesh reduces the hardness of internal fixation devices by wrapping the lower third of the jaw, reducing stress shielding. Moreover, due to its advantages in shape and stress, the 3D printed titanium mesh avoids the trauma of secondary surgery for removal (Liu et al., 2019).

## 5 Conclusion

In summary, the combined use of digital design, 3D printed titanium mesh, double-layer folded fibula, and simultaneous implant placement provides a systematic clinical solution for segmental mandibular defects. The application of full-process digital design in mandibular reconstruction and simultaneous implant placement is of great value. The use of 3D printed titanium mesh, double-layer vascularized fibula, simultaneous implant placement, and the establishment of good occlusal relationships can achieve the goal of early restoration of chewing function and facial appearance, providing clinical practice for precise mandibular reconstruction. However, the broader three-point and long-term effects of this solution still need to be further verified by expanding the patient cohort.

## Data Availability

The original contributions presented in the study are included in the article/Supplementary Material, further inquiries can be directed to the corresponding authors.
